# Supplementary material for: Biological Role of Tumor/Stromal CXCR4-CXCL12-CXCR7 in MITO16A/MaNGO-OV2 Advanced Ovarian Cancer Patients
Source: Cancers (Basel). 2022 Apr 6;14(7):1849. doi: 10.3390/cancers14071849 (PMC8997727; doi:10.3390/cancers14071849)
Supplement: Supplementary file 1 [file cancers-14-01849-s001.zip › cancers-1612933-supplementary.pdf]

Supplementary Material

Supplementary Figures

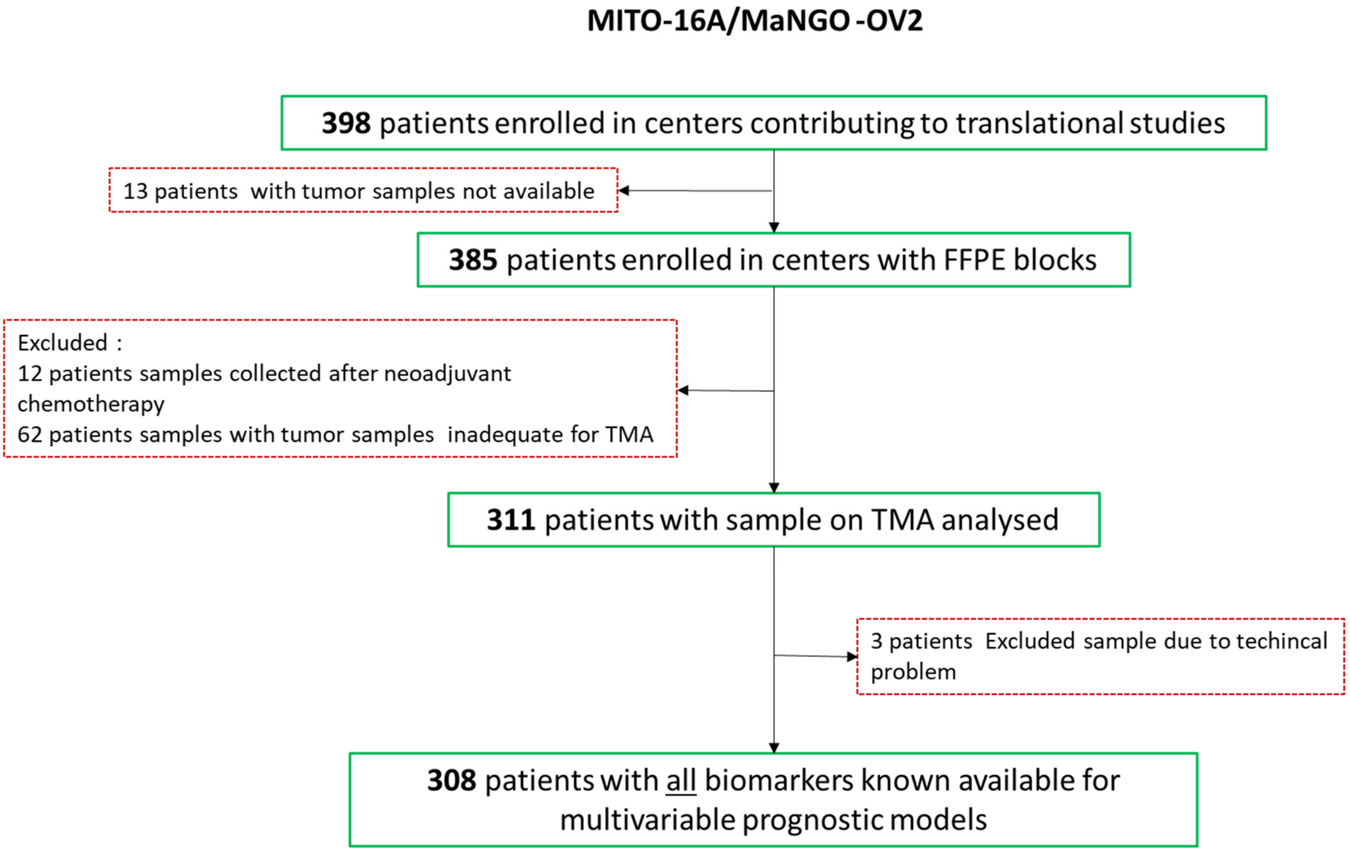

Figure S1. Flow chart of inclusion and exclusion criteria

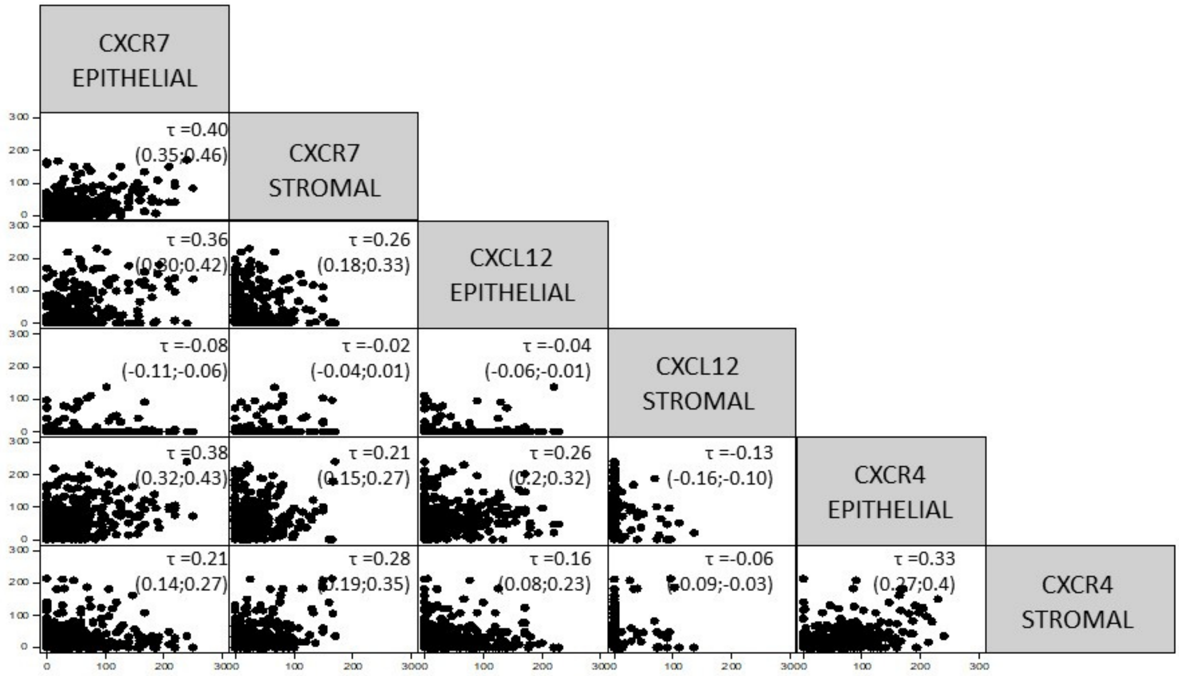

Figure S2. Correlation tested by Kendall test modified for zero inflated Wilcoxon rank test (ZIW) for zero-inflated data with permutations (except for Residual Disease where Kruskal-Wallis zero inflated (ZIKW) was applied).

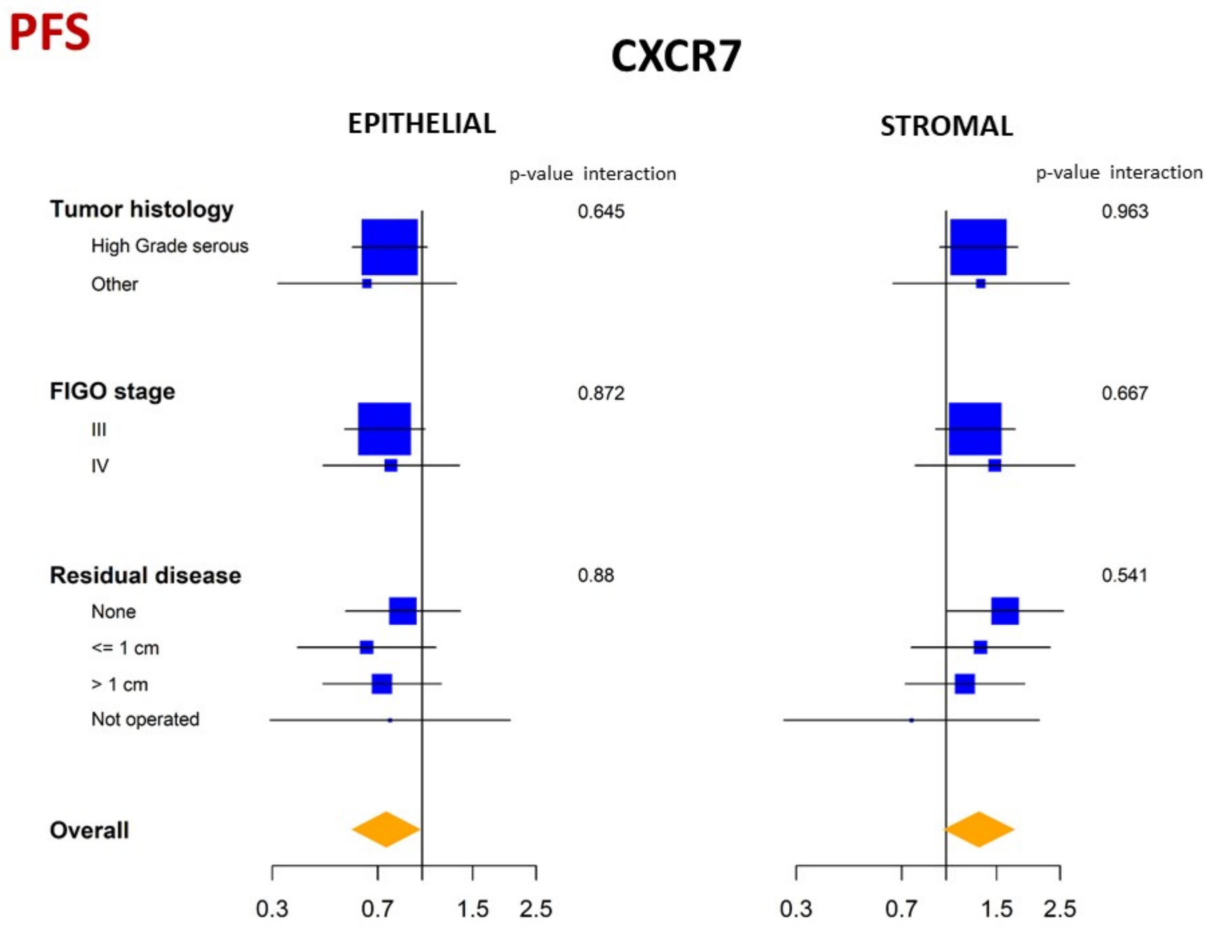

Figure S3. Forest plot of hazard ratios (HR) of progression, and 95% CI, evaluated in different subgroups (Cox proportional hazards model analysis) and interaction comparison between prognostic factor and CXCR7 in epithelial and stromal cells. The x-axis represents the Odds ratio on a log scale with the reference vertical line, Odds ratios (square) and 95% CI (whiskers).

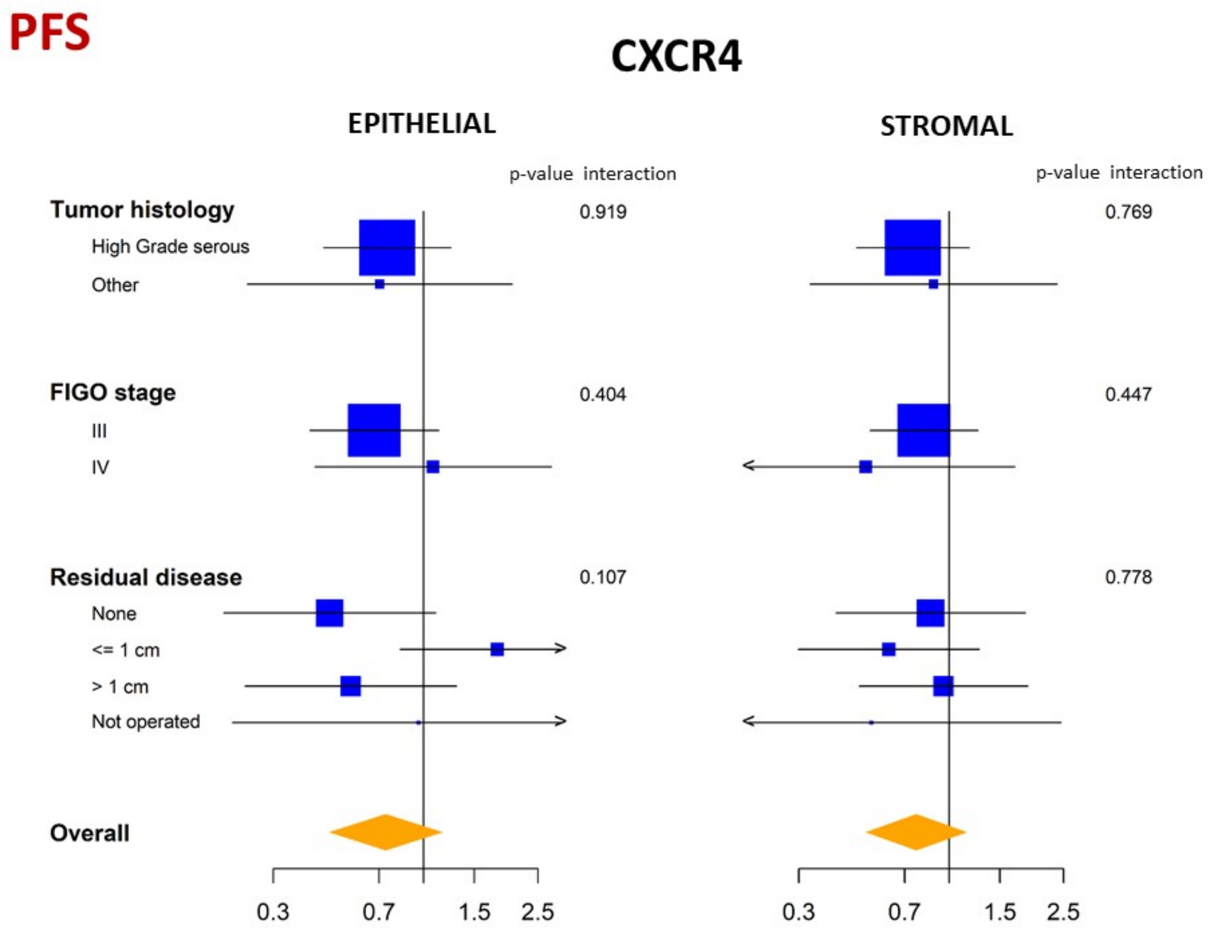

Figure S4. Forest plot of hazard ratios (HR) of progression and 95% CI, evaluated in different subgroups (Cox proportional hazards model analysis) and interaction comparison between prognostic factor and CXCR4 in epithelial and stromal cells. The x-axis represents the Odds ratio on a log scale with the reference vertical line, Odds ratios (square) and 95% CI (whiskers).

Supplementary Tables

Table S1. Patients characteristics in translational analysis

**Age**, median (IQR)	58.8 (49.8-65.9)
**Age elderly**, n (%)	
<65	220 (71.4)
≥65	88 (28.6)
**ECOG performance status**, n(%)	
0	247 (80.2)
1	53 (17.2)
2	8 (2.6)
**Residual disease**, n(%)	
None	130 (42.2)
≤ 1 cm	63 (20.5)
> 1 cm	96 (31.2)
Not operated	19 (6.2)
**FIGO stage**, n(%)	
IIIB	29 (9.4)
IIIC	219 (71.1)
IV	60 (19.5)
**Tumor histology**, n(%)	
High Grade serous	264 (85.7)
Low Grade serous	11 (3.6)
Endometrioid	9 (2.9)
Clear Cell	8 (2.6)
Mucinous	2 (0.6)
Mixed	2 (0.6)
Other	12 (3.9)
**Location**, n(%)	
Primitive	233 (75.6)
Metastatic	75 (24.4)

Table S2. Multivariable analysis of clinical prognostic factors for PFS and OS.

	**PFS (221 events)**			**OS (102 events)**		
	**HR**	**(95% CI)**	**p**	**HR**	**(95% CI)**	**p**
**Age ederly**			0.3346			0.2777
<65y	1	-		1	-	
≥ 65y	0.86	(0.63-1.17)		0.78	(0.49-1.22)	
**ECOG PS**			**0.0326**			0.0595
0	1	-		1	-	
1	1.46	(1.03-2.08)		1.61	(0.98-2.66)	
**FIGO stage**			**<0.001**			**0.0167**
IIIB-IIIC	1	-		1	-	
IV	2.16	(1.56-2.98)		1.75	(1.11-2.78)	
**Residual disease**			**<0.001**			**<0.001**
None	1	-		1	-	
≤ 1 cm	1.47	(1.03-2.11)		1.60	(0.90-2.83)	
> 1 cm	1.86	(1.33-2.60)		2.17	(1.32-3.59)	
Not operated	3.18	(1.84-5.50)		3.10	(1.47-6.54)	
**Tumor histology**			0.1925			0.5092
High grade serous	1	-		1	-	
Other	1.29	(0.88-1.89)		1.21	(0.69-2.11)	
**Centre by N. of enrolled pts**						
<25	1	-	0.9698	1	-	0.1457
>25	0.99	(0.75-1.31)		1.36	(0.90-2.07)	

Table S3. Univariate analysis of biomarkers (continuous and dummy for zero variables) for PFS and OS

	PFS (221 events)			OS (102 events)		
	**HR**	**(95% CI)**	**P**	**HR**	**(95% CI)**	**P**
**CXCR7:**						
**Continuous variables**						
Epithelial	0.93	(0.86-1.01)	0.106	0.99	(0.88-1.10)	0.821
Stromal	1.00	(0.89-1.12)	0.980	1.02	(0.87-1.20)	0.797
**Dummy variables for zero**						
Epithelial	0.74	(0.49-1.11)	0.148	0.86	(0.48-1.55)	0.623
Stromal	0.94	(0.65-1.35)	0.728	0.99	(0.58-1.69)	0.969
**CXCL12:**						
**Continuous variables**						
Epithelial	1.03	(0.95-1.11)	0.500	1.08	(0.96-1.21)	0.190
Stromal	1.10	(0.81-1.49)	0.535	0.94	(0.56-1.57)	0.810
**Dummy variables for zero**						
Epithelial	0.85	(0.61-1.2)	0.366	0.92	(0.56-1.52)	0.745
Stromal	1.57	(0.8-3.06)	0.186	1.45	(0.53-4.00)	0.470
**CXCR4:**						
**Continuous variables**						
Epithelial	0.94	(0.87-1.02)	0.143	0.98	(0.87-1.09)	0.678
Stromal	0.99	(0.89-1.11)	0.910	0.96	(0.82-1.12)	0.579
**Dummy variables for zero**						
Epithelial	0.69	(0.41-1.18)	0.176	0.82	(0.37-1.80)	0.619
Stromal	0.95	(0.67-1.35)	0.783	0.87	(0.52-1.45)	0.594

Table S4. Analysis of biomarkers best cut-off adjusted for clinical characteristics for PFS and OS using Shrunken coefficients.

	**Shrunken coefficients**					
	**Progression Free Survival**			**Overall Survival**		
	**HR**	**(95% CI)**	**p**	**HR**	**(95% CI)**	**p**
**CXCR7 Epithelial**						
> 36.7	0.80	(0.37-1.76)	0.586	1.01	(0.16-6.29)	0.992
**CXCR7 Stromal**						
> 20.0	1.20	(0.47-3.08)	0.706	1.26	(0.24-6.51)	0.784
**CXCL12Epithelial**						
> 21.7	1.24	(0.62-2.46)	0.546	1.53	(0.61-3.81)	0.361
**CXCL12 Stromal**						
> 6.7	0.92	(0.15-5.63)	0.930	0.81	(0.1-6.83)	0.843
**CXCR4 Epithelial**						
> 130.0	0.88	(0.35-2.27)	0.799	0.87	(0.16-4.69)	0.871
**CXCR4 Stromal**						
> 65.0	0.91	(0.37-2.19)	0.827	0.98	(0.17-5.62)	0.983

Table S5. Analysis of biomarkers (continuous and dummy for zero variables) adjusted for clinical characteristics for PFS and OS

	PFS (221 events)			OS (102 events)		
	**HR**	**(95% CI)**	**P**	**HR**	**(95% CI)**	**P**
**CXCR7:**						
**Continuous variables**						
Epithelial	0.94	(0.85-1.03)	0.1617	0.98	(0.86-1.10)	0.6976
Stromal	1.05	(0.93-1.20)	0.4326	1.09	(0.91-1.30)	0.3613
**Dummy variables for zero**						
Epithelial	0.97	(0.63-1.52)	0.9064	1.14	(0.60-2.18)	0.6892
Stromal	0.88	(0.60-1.30)	0.5214	0.92	(0.52-1.64)	0.7824
**CXCL12:**						
**Continuous variables**						
Epithelial	1.02	(0.94-1.11)	0.6341	1.07	(0.94-1.21)	0.2935
Stromal	1.16	(0.85-1.57)	0.3450	1.08	(0.64-1.82)	0.7771
**Dummy variables for zero**						
Epithelial	0.86	(0.59-1.27)	0.4561	0.84	(0.47-1.50)	0.5566
Stromal	1.61	(0.82-3.15)	0.1647	1.82	(0.64-5.20)	0.2632
**CXCR4:**						
**Continuous variables**						
Epithelial	0.98	(0.90-1.06)	0.5906	0.99	(0.86-1.13)	0.8703
Stromal	0.97	(0.86-1.10)	0.6411	0.97	(0.81-1.18)	0.7930
**Dummy variables for zero**						
Epithelial	0.76	(0.43-1.34)	0.3483	0.82	(0.34-1.96)	0.6526
Stromal	0.94	(0.65-1.36)	0.7379	0.90	(0.52-1.55)	0.7078

Table S6. CXCR4, CXCL12 and CXCR7 expression and Response to treatment.

Variable	Response (No) N = 48	Response (Yes) N = 146	P-Value
**CXCR7:**			
Epithelial	20 (0-60)	40 (10-76)	**0.047**
Stromal	15 (0-40)	20 (0-43)	0.527
**CXCL12:**			
Epithelial	29 (0-83)	13 (0-56)	0.193
Stromal	0 (0-0)	0 (0-0)	0.647
**CXCR4:**			
Epithelial	43 (9-67)	73 (38-93)	**<0.001**
Stromal	10 (0-31)	20 (0-43)	0.072

Values are median (IQR); p-values were calculated with Wilcoxon rank test for zero-inflated data.
